# Multispectral Drone Imagery and SRGAN for Rapid Phenotypic Mapping of Individual Chinese Cabbage Plants

**DOI:** 10.34133/plantphenomics.0007

**Published:** 2022-12-19

**Authors:** Jun Zhang, Xinxin Wang, Jingyan Liu, Dongfang Zhang, Yin Lu, Yuhong Zhou, Lei Sun, Shenglin Hou, Xiaofei Fan, Shuxing Shen, Jianjun Zhao

**Affiliations:** ^1^ State Key Laboratory of North China Crop Improvement and Regulation, Hebei Agricultural University, 071000 Baoding, China.; ^2^ College of Mechanical and Electrical Engineering, Hebei Agricultural University, 071000 Baoding, China.; ^3^ Mountain Area Research Institute, Hebei Agricultural University, 071001 Baoding, China.; ^4^ College of Horticulture, Hebei Agricultural University, 071000 Baoding, China.; ^5^ Hebei Academy of Agriculture and Forestry Sciences, 050000 Shijiazhuang, China.

## Abstract

The phenotypic parameters of crop plants can be evaluated accurately and quickly using an unmanned aerial vehicle (UAV) equipped with imaging equipment. In this study, hundreds of images of Chinese cabbage (*Brassica rapa* L. ssp. *pekinensis*) germplasm resources were collected with a low-cost UAV system and used to estimate cabbage width, length, and relative chlorophyll content (soil plant analysis development [SPAD] value). The super-resolution generative adversarial network (SRGAN) was used to improve the resolution of the original image, and the semantic segmentation network Unity Networking (UNet) was used to process images for the segmentation of each individual Chinese cabbage. Finally, the actual length and width were calculated on the basis of the pixel value of the individual cabbage and the ground sampling distance. The SPAD value of Chinese cabbage was also analyzed on the basis of an RGB image of a single cabbage after background removal. After comparison of various models, the model in which visible images were enhanced with SRGAN showed the best performance. With the validation set and the UNet model, the segmentation accuracy was 94.43%. For Chinese cabbage dimensions, the model was better at estimating length than width. The *R*^2^ of the visible-band model with images enhanced using SRGAN was greater than 0.84. For SPAD prediction, the *R*^2^ of the model with images enhanced with SRGAN was greater than 0.78. The root mean square errors of the 3 semantic segmentation network models were all less than 2.18. The results showed that the width, length, and SPAD value of Chinese cabbage predicted using UAV imaging were comparable to those obtained from manual measurements in the field. Overall, this research demonstrates not only that UAVs are useful for acquiring quantitative phenotypic data on Chinese cabbage but also that a regression model can provide reliable SPAD predictions. This approach offers a reliable and convenient phenotyping tool for the investigation of Chinese cabbage breeding traits.

## Introduction

With the development of informatization and digitalization technologies, remote sensing has become an important approach for obtaining farmland information in precision agriculture. It is also an important data source for plot area calculation, crop species identification, and growth analysis [1]. In recent years, plant phenotyping technologies have played important roles in plant research and agriculture [2–6]. Previous studies have focused mainly on yield prediction in cereals such as maize (*Zea mays* L.) [7] and wheat (*Triticum aestivum* L.) [8] or fruit such as grapes (*Vitis vinifera* L.) [9].

As remote sensing has become an important data source for yield prediction, aboveground prediction, and nutrient management, researchers have used unmanned aerial vehicles (UAVs) loaded with multispectral imaging equipment to study crop density. Wilke et al. [10] established a linear regression between manually counted barley and wheat density and counts acquired using a UAV. Remote sensing imaging combined with machine learning has been used for fruit yield prediction, e.g., in grapes [9]. Ground reference yield datasets have been compared with yields predicted using UAVs. UAVs combined with multispectral images have also been used to estimate the aboveground biomass of maize, showing that this approach could effectively improve the accuracy, stability, and universality of biomass prediction [11,12]. Over time, remote sensing is clearly becoming a more and more mature technology for yield and biomass estimation [13]. In addition, because nutrients are critical for crop growth [14], UAVs combined with multispectral sensors can make use of highly accurate predictive models to quickly obtain information on physiological parameters in the field, e.g., chlorophyll content [15,16]. Monitoring of vegetation indices with a UAV during the crop cycle promotes agile decision-making about management practices and provides information on crop deficiencies in nutrients, such as nitrogen (N) [17,18]. Thus, UAV mapping can serve as an efficient tool for field management by enabling high-throughput monitoring of crop growth.

Deep learning is becoming increasingly popular in remote sensing [17,19]. For example, researchers proposed a hybrid convolution neural network (CNN) model to detect maize leaves infested by fall armyworms. The proposed CNN model was designed to combine the benefits of 2 individual models (VGG16 and InceptionV3), thus reducing training time and achieving superior accuracy [20]. In addition, crop researchers reported that deep learning models were better than traditional machine learning methods for yield and trait prediction [17,21]. Zhou et al. [22] demonstrated the feasibility of deep learning combined with UAV images for soybean breeding and showed that a UAV combined with deep learning produced good classification performance for estimation of flood-induced soybean injuries. These results indicated that the proposed method had great promise for soybean breeding, and other researchers have also shown that rapid methods for measuring crop responses are very important for plant breeding [13,23]. Deep learning models have therefore been incorporated into the present experiment.

Chinese cabbage (*Brassica rapa* L. ssp. *pekinensis*) is one of the most important vegetables in Asia [24]. As the origin and center of genetic diversity of nonheading Chinese cabbage, China has enormously rich germplasm resources and many local varieties [25,26]. Phenotypic identification of Chinese cabbage is important for breeding, but traditional methods of measuring Chinese cabbage phenotypes rely heavily on manual evaluation and measurement, which are highly subjective and time-consuming [27]. Drones combined with deep learning have mainly been used for field crops such as maize [7] and wheat [28]. We therefore sought to determine whether remote sensing technology combined with deep learning would show similar performance in Chinese cabbage. To that end, we applied 2 deep learning techniques, super-resolution generative adversarial network (SRGAN) for enhancing drone image resolution [29] and the improved semantic segmentation model Unity Networking (UNet) for separating individual cabbages from the complex background [30].

Chinese cabbage is a dwarf plant that typically requires high-resolution drone images for trait investigation during breeding; SRGAN was therefore introduced in this research to improve prediction accuracy. SRGAN has been widely used in pathology and electromagnetics [31–34] but rarely in agriculture. Medical images (computer tomography images, magnetic resonance images, etc.) have been enhanced using SRGAN, and this approach has been shown to generate high-quality images [31], as well as a superior peak signal-to-noise ratio (PSNR) and structural similarity score (SSIM) [35]. In addition, some researchers have used SRGAN to improve the resolution of weather images, thereby increasing the accuracy of weather prediction [36]. In a relevant study on the estimation of maize plant density, an advanced super-resolution method based on the generative adversarial network (GAN) was applied to native low-resolution images (ground sampling distance [GSD] ≈ 0.6 cm), and the relative root mean square error (rRMSE) dropped from 0.48 to 0.22 [37]. This was still far below the performance achieved with native high-resolution images (GSD ≈ 0.3 cm), but the large memory consumption of high-resolution images was not conducive to the deep learning framework.

Here, we used SRGAN to improve the resolution of drone images of Chinese cabbage. A UNet model was then used to segment the target plants from the background. Three deep learning models for remote sensing images were established: a multispectral image model, an RGB image model without resolution enhancement, and an RGB image model with resolution enhancement. The ultimate goal was to find the optimal model for predicting Chinese cabbage phenotype data, specifically width, length, and soil plant analysis development (SPAD) value.

## Materials and Methods

Drone images of Chinese cabbage in the planting field were collected, and 3 deep learning predictive models based on UAV images were established using multispectral images, visible images, and visible images with enhanced resolution. The optimal model was selected by comparison and could then be used to achieve rapid and nondestructive prediction of Chinese cabbage phenotype data.

### Study site and samples

**Fig. 1.**
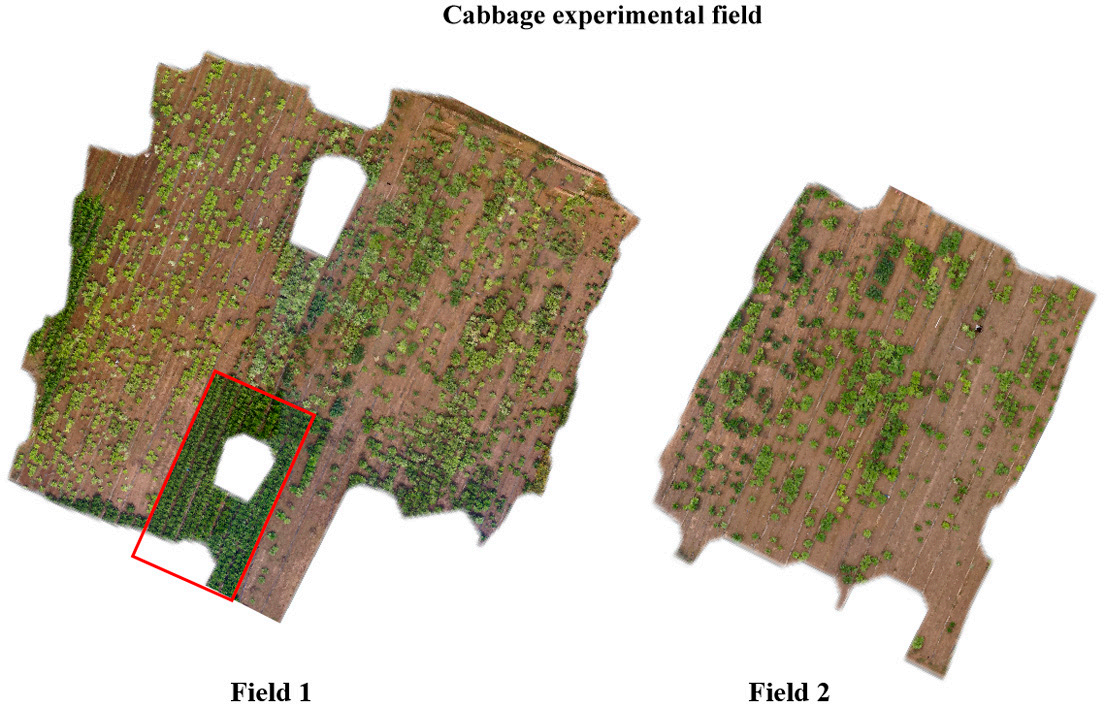
Map of the study site. The Chinese cabbages outlined in the red box were the commercial varieties. The other Chinese cabbages were the main subjects of the present research. (For interpretation of the references to color in this figure legend, the reader is referred to the web version of this article.)

This experiment was carried out at Malan Farm (Xinji County, Hebei Province, China), an experimental field site of Hebei Agricultural University located at 37°94′ East and 115°20′ North (Fig. 1). Conventional planting methods were used. The plants with high uniformity were commercial Chinese cabbage varieties (shown in the red box in Fig. 1), whereas the materials used in this study were self-bred, noncommercial Chinese cabbage varieties. There were more than 2,000 individual cabbage plants, including 274 varieties of introduced materials and mutant materials.

### Acquisition of multispectral images and phenotype data

The multispectral images were captured with a lightweight, portable drone imagery platform, DJI Phantom 4 Pro. The aircraft weighed 1,280 g; it had a 4,480-mAh battery and a maximum flight speed of 15 m/s for 25 min. The multispectral camera (Model No. FC6360_5.7) was used to acquire 5-band images that contained the visible RGB bands, a red edge band at 730 to 770 nm, and a near-infrared band at 780 to 890 nm; it was equipped with a perspective lens. The height of the camera sensor was 4.08 mm, and its width was 5.02 mm. The camera had a focal length of 5.74 mm, a pixel size of 3 μm, and a sensor size of 1/2.9 in.. The UAV was flown at an altitude of 10 m with a spatial resolution of 5 cm. To minimize the influence of sunlight, the imaging angle was set to 108°, parallel to the angle of the sunlight, and the repetition rate of image capture was 40%.

A portion of the cabbage plants that were easy to identify from the UAV images were selected for manual in situ measurement of length and width. At the same time, the relative chlorophyll contents of some cabbage plants were measured using a SPAD meter (502 Plus, KONICA MINOLTA). These data were used for evaluation and verification of the newly established predictive models.

### Image preprocessing

#### Image stitching and synthesis

DJI Terra software was used to stitch together the collected images and acquire an image of the entire experimental field, with position and orientation system points. At the same time, we obtained an RGB stitched image and stitched gray-scale images of the 5 bands R, G, B, Red edge, and near-infrared. ArcMap software was used to process the stitched images, and a composite image of the multispectral and visible images was obtained. At the same time, the software was also used to mark each cabbage in the complete stitched image. To facilitate the training of the UNet semantic segmentation model, the 5-channel composite image and the RGB image were made to correspond to the label dataset; that is, each cabbage was given the same label in each image.

#### Image cropping

The image cropping process was implemented in Python (version 3.6.13). The size of the cropped images was set to 608 × 608 pixels, and the repetition rate of the cropped images was set to zero. That is, the original image was cropped from top to bottom and left to right in order. The visible image, multispectral image, and labeled image were cropped at the same time, and 1,924 images were obtained for each category. The cropped images were then used for resolution enhancement and image segmentation.

### Data division and image data augmentation

The data were divided into a training set, test set, and validation set at a ratio of 8:1:1. The training set was augmented by image data enhancement using geometric transformation, vertical and horizontal inversion, and diagonal mirroring to produce more multispectral and RGB images, with each of these images assigned to its corresponding label. The validation set was used for hyperparameter tuning, and the test set was used for the model evaluation.

### Resolution enhancement

To improve photo resolution, SRGAN [29] was used to perform super-resolution enhancement on the collected images. SRGAN consists of generator network (G Network) and discriminant network (D Network). SRGAN takes a low-resolution image as input and generates a high-resolution image. The high-resolution image was generated by G Network through the low-resolution image, and whether the image was generated by G Network or the original image in the database was judged by D Network. Perceptual loss and adversarial loss in SRGAN were used to enhance the realism of the resulting high-resolution images. The SRGAN generation network consisted of 3 parts, and a pretrained model was used in this study. The first part was a convolutional layer with activation layer (the function of rectified linear unit) following the input layer of the low-resolution images. The second part was composed of a series of residual blocks, each of which included 2 convolutional layers, 2 normalization layers, 2 rectified linear unit activation layers, and a residual edge. The third part was for up sampling: the height and width of the original image were increased four-fold relative to the original image, and the resolution of the image was then enhanced. Therefore, the first 2 parts of the network were used for feature extraction, and the third part was used for resolution improvement. The SRGAN discriminative network consisted of repeated convolution layers, activation layers, and normalization layers. The training process of the network was completed in 2 steps, generator training and discriminator training. In each training step, the discriminator was trained first and then the generator was trained. The SSIM index from 0 to 1 was used to measure the similarity of the 2 images, and a larger value indicated better performance of the model. To measure image quality after processing, PSNR was used as an objective criterion for evaluating the resolution of the enhanced images.

### Image segmentation

**Fig. 2.**
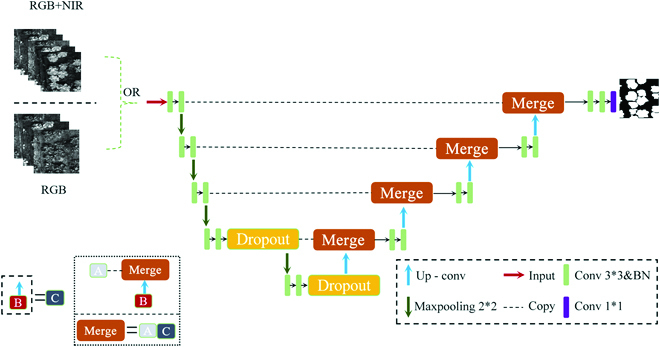
The framework of the deep learning segmentation model. In this experiment, the input could be multispectral images, RGB images, or RGB images with enhanced resolution. The output was the predicted labels, which were used to segment the Chinese cabbage images. In this figure, conv represents the convolution layer, up-conv represents the layer of up-sampling and then convolution, maxpooling represents the maximum pooling layer, copy represents the copy of the convolutional layer, merge represents the merge of the 2-function layer, and dropout represents the dropout layer.

The improved UNet framework for deep learning was used to segment the Chinese cabbages from the background field of the drone images. The deep learning framework is shown in Fig. 2. The input for the semantic segmentation model was a 5-channel multispectral image or an RGB image. The model was developed with Keras (version 2.1.5), and the geospatial data abstraction library was used to read the multiband images. The labels were one-hot encoded for image preprocessing; that is, each class of the label was individually turned into a layer composed of 0 and 1. The loss function used in this study was the function of categorical cross entropy loss. As for the calculation of segmentation accuracy, the accuracy was calculated on the basis of the label of the segmented images with the original labels. According to the data generator, training data and validation data were generated at the rate of batch size. The model was trained using the fit_generator function in Keras, and the training data was the data generated by the generator [30].

### Calculation and evaluation

**Fig. 3.**
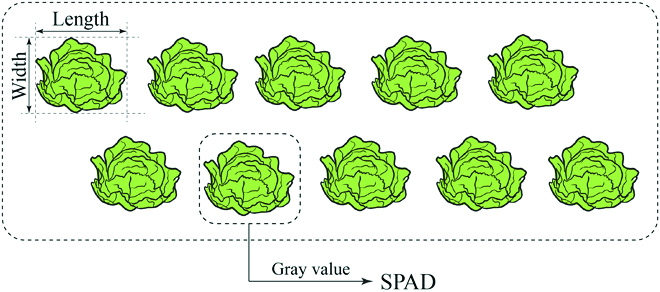
A schematic diagram of the arrangement of Chinese cabbages and the prediction of 3 phenotypic parameters. The length and width of the plants were estimated with pixel numbers, and the SPAD value was predicted from the gray value.

To illustrate the prediction of the 3 phenotypic parameters (length, width, and SPAD value) in more detail, a schematic diagram is presented in Fig. 3. The prediction label obtained by UNet was superimposed with the original image, and the cabbages were then separated from the ground in the image. The function of get the properties of region (regionprops) was used to acquire the corresponding region of each cabbage, marked with a rectangle and a number. Then, the width, length and SPAD of each cabbage were calculated.

#### Calculation of width and length

GSD was used to calculate the actual widths and lengths. The relative navigation height *H* was set to 10 m, and GSD was calculated with Eq. 1. *a* represents the pixel size, and *f* represents the focal length.

$$\mathrm{GSD}=H\times \frac{a}{f}$$ (1)

The maximum resolution of each captured image was 1,600 × 1,300 (4:3.25), and the actual length (*L*), width (*W*), and area (*S*) of the captured images were therefore calculated with Eqs. 2 to 4.

L=1600×GSD (2)

W=1300×GSD (3)

S=L×W (4)

The actual length of each acquired image was ~8.36 m, the width was ~6.79 m, and the size of each pixel was ~5.23 mm. The obtained images were 608 × 608 pixels in size after stitching and clipping, and their actual size was therefore 3.18 × 3.18 m^2^. The actual width and length of each Chinese cabbage were then calculated on the basis of the GSD.

#### SPAD prediction

We established a multiple linear regression (MLR) model to predict the SPAD value of each Chinese cabbage using Unscrambler X software (Version 10.4, CAMO Software Inc., USA). The model input was the value of the 3 RGB channels and the value of the G channel, and the output was the SPAD value estimate.

#### Evaluation of the predicted parameters

The coefficient of determination (*R* squared, *R*^2^), root mean square percentage error (RMSPE), and root mean square error (RMSE) were used in this study to evaluate the prediction performance for each phenotypic measurement (width, length, and SPAD value). These evaluation parameters were calculated with Eqs. 5 to 7.

$$R^{2}=\frac{\sum {\left({\hat{y}}_{i}-\bar{y}\right)}^{2}}{\sum {\left(y_{i}-\bar{y}\right)}^{2}}\;$$ (5)

$$\text{RMSPE}=\sqrt{\frac{1}{n}\sum_{i=1}^{n}\frac{{\left(y_{i}-{\hat{y}}_{i}\right)}^{2}}{y_{i}}}$$ (6)

$$\text{RMSE}=\sqrt{\frac{\sum_{i=1}^{n}{\left(y_{i}-{\hat{y}}_{i}\right)}^{2}}{n}}\;$$ (7)

where *y_i_* represents the actual value of the *i*th sample, ${\hat{y}}_{i}$ represents the predicted value of the *i*th sample, $\bar{y}$ represents the average value of all samples, and *n* represents the number of samples.

## Results

### Image labeling

**Fig. 4.**
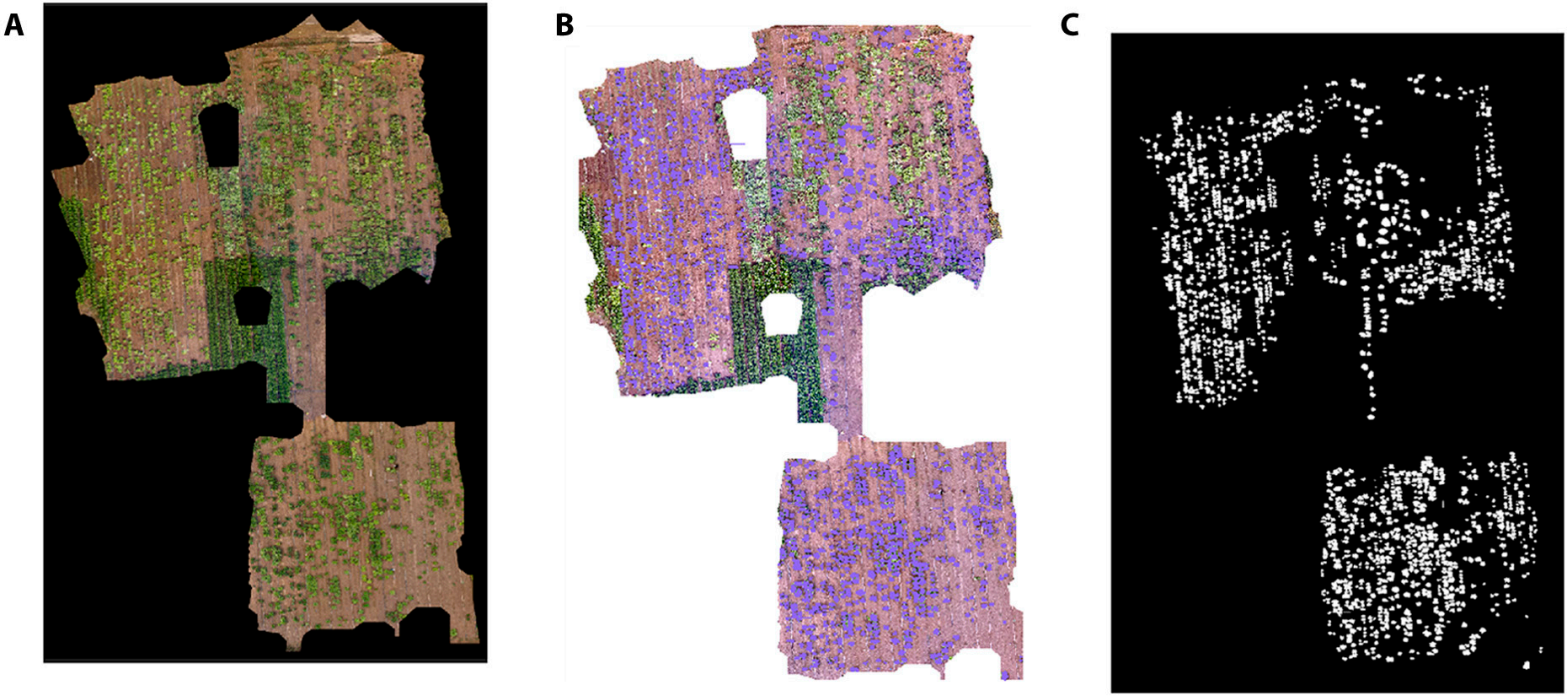
Steps used to generate labels in ArcMap. (A) The original image. (B) Labeling the Chinese cabbages one by one. (C) The labeled figure.

We created the project using ArcMap and marked each plant one by one. The label generation process for the complete image is shown in Fig. 4. The samples in the middle of the field were commercial varieties that were used to protect the self-cultivated varieties, and their uniformity made them easy to distinguish. There was no marking or analysis of the commercial Chinese cabbages.

### Resolution enhancement by SRGAN

**Fig. 5.**
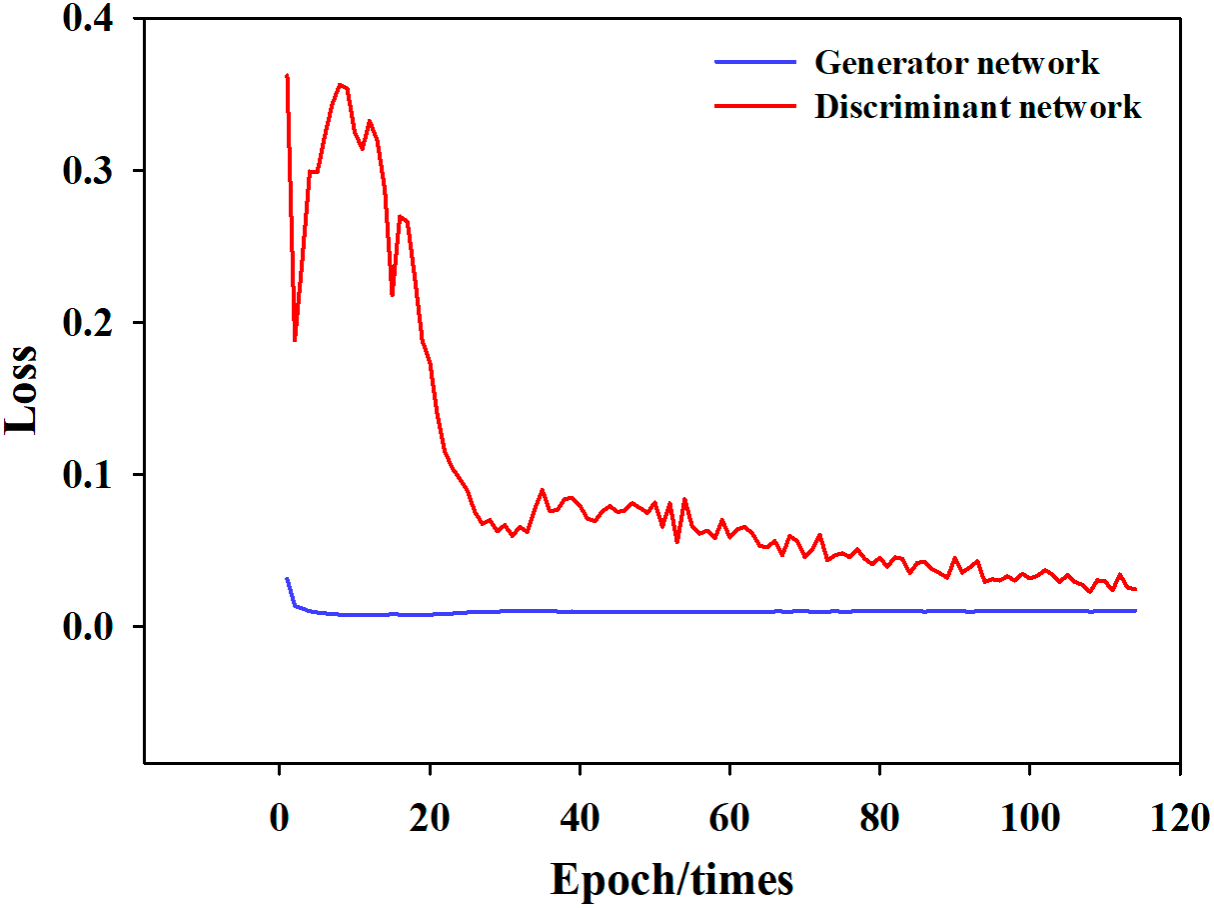
G Network loss and D Network loss.

**Fig. 6.**
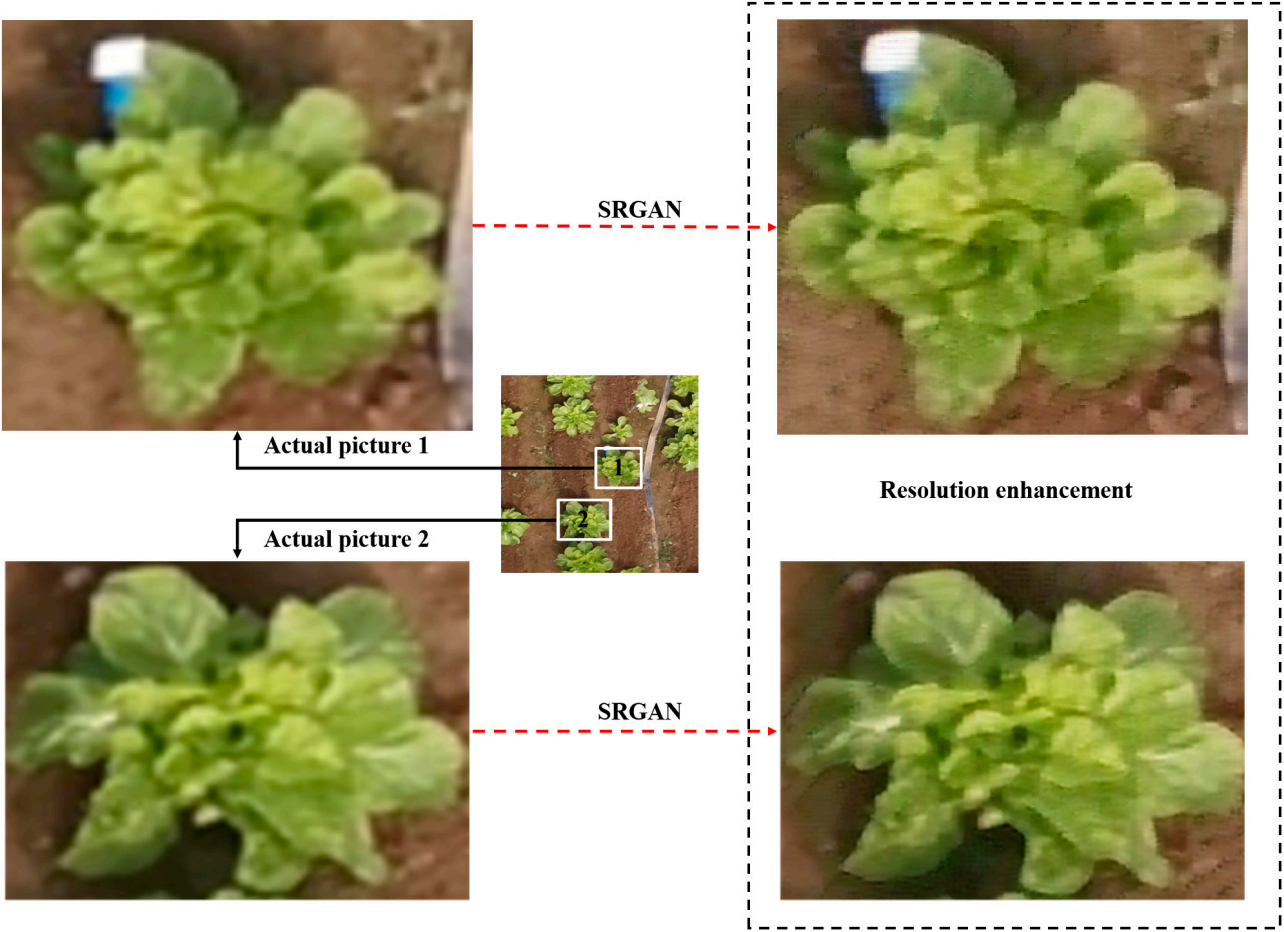
The performance of the SRGAN resolution enhancement. Two individual Chinese cabbages are shown as examples; on the left is the actual image, and on the right is the image with SRGAN resolution enhancement.

To train the SRGAN model, the classifier was trained first, followed by the generator. When a low-resolution image was fed into the generation model, a false high-resolution image was obtained. The generator loss could be determined by comparing the discrimination result obtained from the false high-resolution image with the actual image. The real high-resolution image and the fake high-resolution image were passed into the VGG network, the features of the 2 images were obtained, and the discriminant loss was obtained by comparing the features of the 2 images. The G Network loss and D Network loss are shown in Fig. 5. The evaluation indices of the final model were PSNR = 27.8 and SSIM = 0.715. The performance of the SRGAN is shown in Fig. 6, demonstrating that resolution was improved by the model.

### UNet

**Fig. 7.**
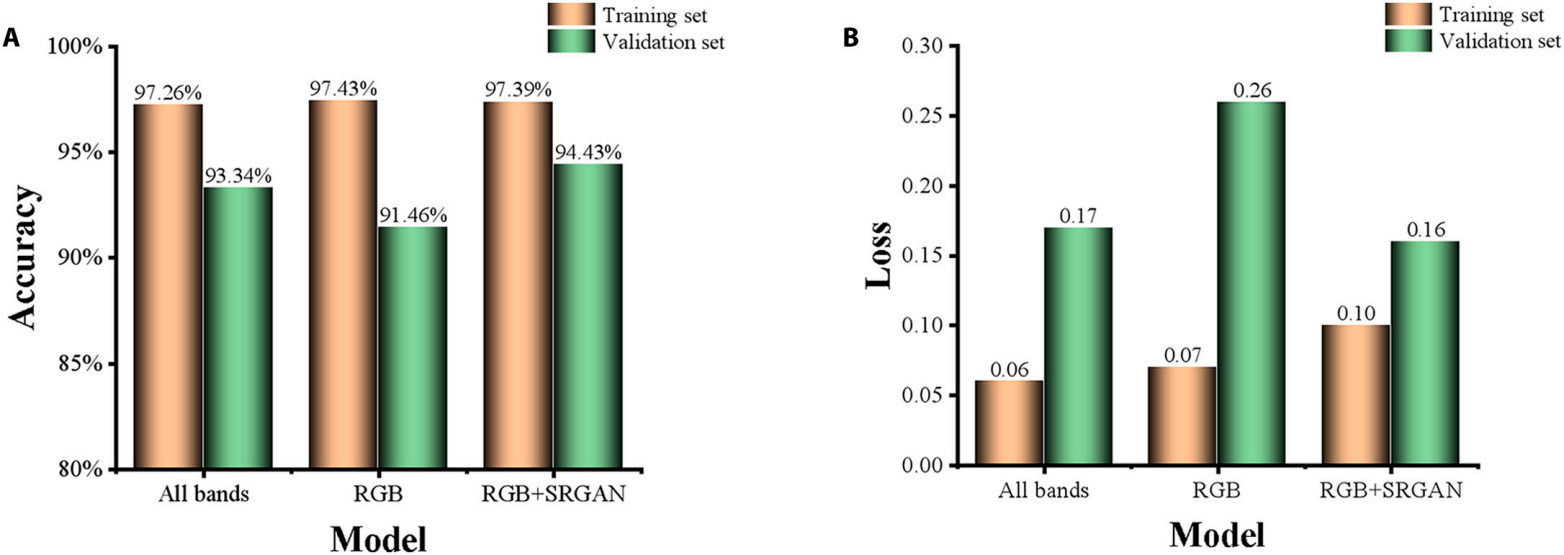
The accuracy and loss of the 3 models for (A) the accuracy and (B) the loss. For the validation set, the model with RGB images enhanced by SRGAN showed the best performance, with the highest accuracy and the smallest loss.

The input to the UNet model was a multispectral image, an RGB image, or an RGB image with resolution enhancement. Three deep learning models were established with the UNet model. In order to evaluate the models, the validation set was used to optimize the model. The models used 10 convolution layers, the batch processing parameter was set to 2, and the initial learning rate was set to 1 × 10^−5^. The epoch of the models was 120. The models did not include pretrained weights, as these were not required for the UNet model in this study. The accuracy and loss of the models are shown in Fig. 7.

### The prediction performance of UNet

#### Width and length

**Fig. 8.**
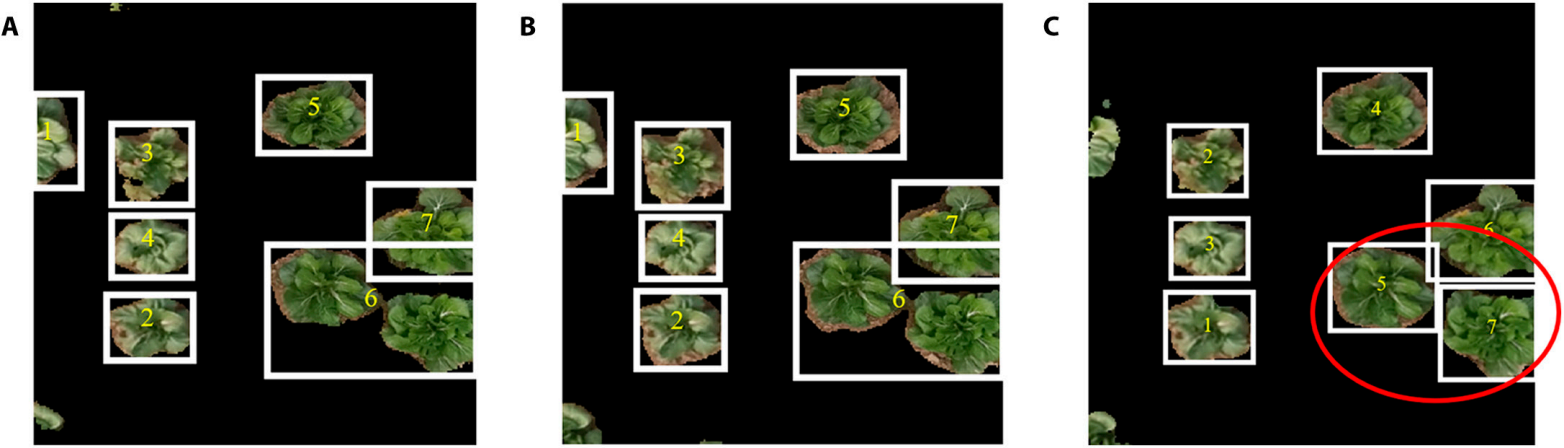
The prediction performance for the validation set. (A) The result using all bands. (B) The result using the RGB bands. (C) The result using the RGB bands with SRGAN resolution enhancement.

The prediction performance of the 3 models for the validation set is shown in Fig. 8. The segmented result of the RGB image combined with the SRGAN model was the best, as all individual plants were successfully located and segmented. The 3 models were also used to predict the actual cabbage widths and lengths using GSD.

**Fig. 9.**
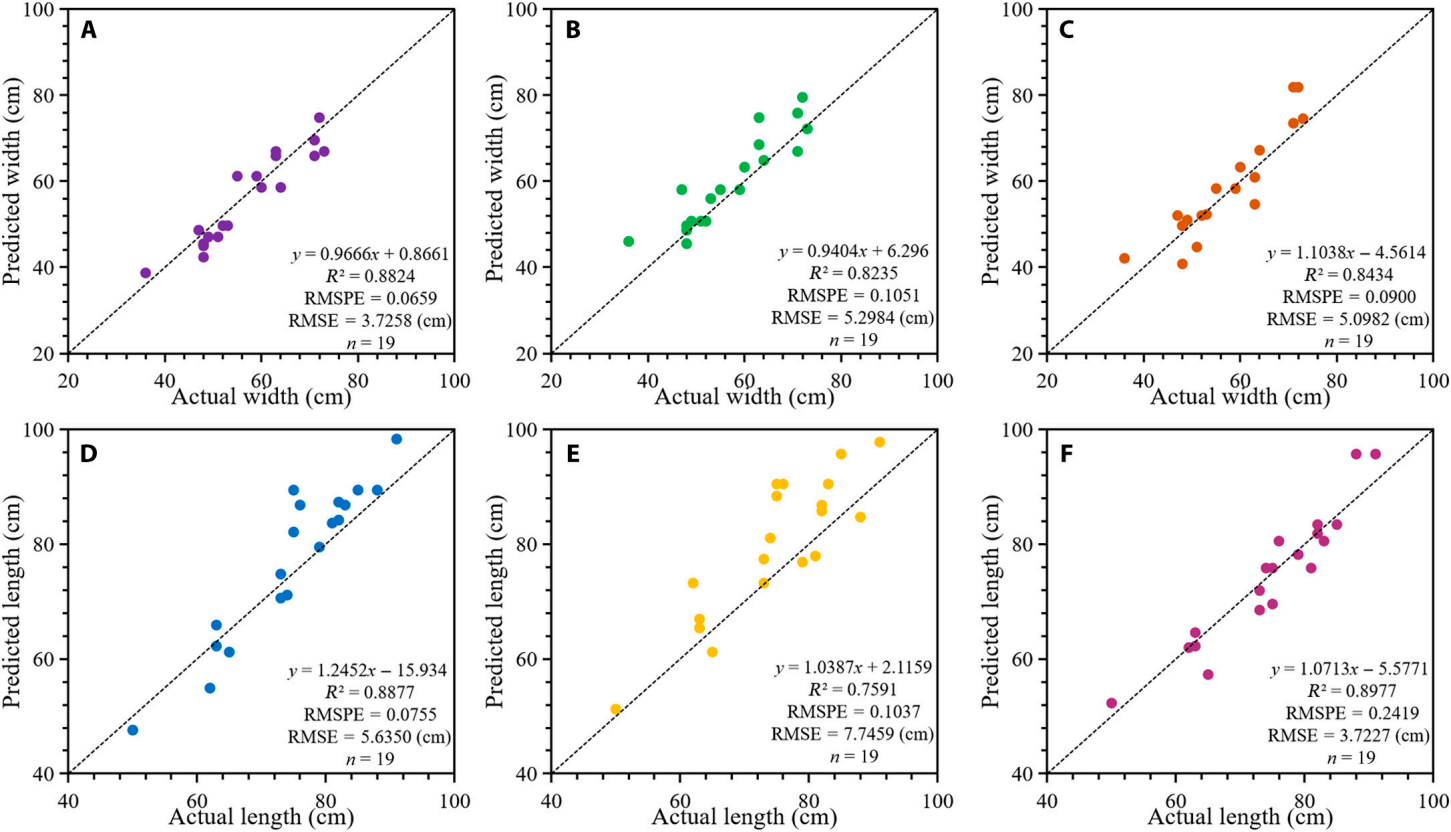
The fitting performance of the 3 UNet models. (A) Actual vs. predicted width for the multispectral image model. (B) Actual vs. predicted width for the model with RGB images and no resolution enhancement. (C) Actual vs. predicted width for the model with RGB images and resolution enhancement. (D) Actual vs. predicted length for the multispectral image model. (E) Actual vs. predicted length for the model with RGB images and no resolution enhancement. (F) Actual vs. predicted length for the model with RGB images and resolution enhancement.

**Fig. 10.**
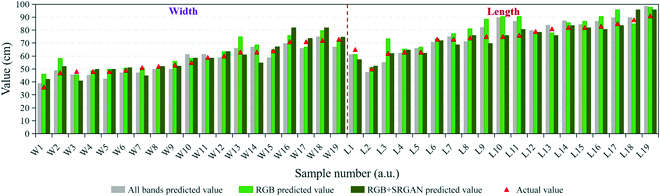
Comparisons of the actual values and predicted values from the 3 models. The red triangles represent the actual values, and the cluster histograms represent the predicted values from the 3 models. (For interpretation of the references to color in this figure legend, the reader is referred to the web version of this article.) a.u., arbitrary units.

The predicted widths and lengths were calculated on the basis of GSD for the 19 samples that we physically measured on site. Figure 9 shows the correlation between the actual and predicted data, demonstrating the good performance of the length and width predictions from the remote sensing images combined with the deep learning model. Figure 10 shows the actual measured values and predicted values of the 3 UNet models; it indicates that the predicted values of the RGB image model without resolution enhancement had a larger error than the measured values.

#### SPAD prediction

**Fig. 11.**
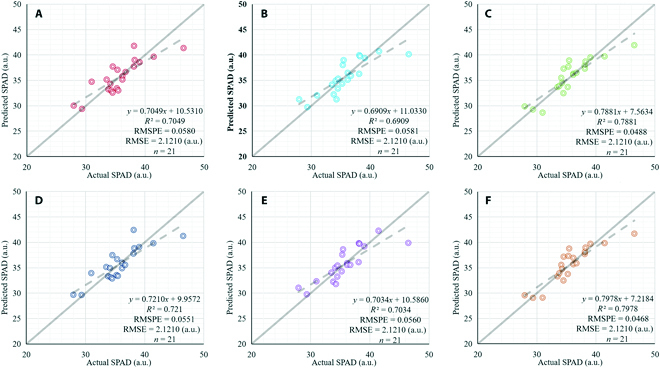
The performance of the SPAD prediction. (A) Actual vs. predicted SPAD values for the multispectral image model based on the G channel. (B) Actual vs. predicted SPAD values for the RGB model based on the G channel. (C) Actual vs. predicted SPAD values for the RGB model with resolution enhancement based on the G channel. (D) Actual vs. predicted SPAD values for the multispectral image model based on the 3 RGB channels. (E) Actual vs. predicted SPAD values for the RGB model based on the 3 RGB channels. (F) Actual vs. predicted SPAD values for the RGB model with resolution enhancement based on the 3 RGB channels.

With the model training, the values of the 3 channels in the visible bands were obtained and were then used to predict the SPAD values. Because green leaves reflect green light [27], MLR models of SPAD prediction were established using both the value of the G channel and the value of the 3 RGB channels. The 2 models based on different inputs were then compared. Figure 11 shows the correlations of predicted versus actual SPAD values for the 21 samples whose SPAD values were measured on site. The results indicated that the MLR model based on 3 channels was better than the MLR model based only on the G channel. Furthermore, the RGB 3-channel model that incorporated SRGAN resolution enhancement had the best performance of the 3 segmentation methods.

## Discussion

The research objects of this study were special, self-bred Chinese cabbage varieties. Images of the cabbage field were spliced using the longitude and latitude of the UAV location. To avoid internal problems in model operation, we cropped the complete stitched image into images of 608 × 608 pixels each, and the resolution of the images was then improved with SRGAN. The UNet deep learning model was trained on the cropped images, establishing models for RGB images with the original resolution, RGB images with enhanced resolution, and multispectral images. We observed that the segmentation accuracy of the RGB images enhanced with SRGAN was higher than that of the other 2 image types, and the loss was lower as well; in addition, higher accuracy was achieved with the multispectral images than with the RGB images without resolution enhancement (Fig. 7). Su et al. [38] used multispectral remote sensing imaging combined with a semantic segmentation model based on deep learning to monitor rice lodging, and they reported an accuracy of 97.30%. The UNet model has also been used in weed identification, and the collected UAV images were also spliced and cropped into images of the same size [39]. In that case, the identification accuracy for weeds was 89.95%. The specific objective of these studies was to identify a whole group of species. In the present study, the segmentation accuracy of Chinese cabbage using a semantic segmentation network was 94.43%, slightly lower than that for rice, above 2.87%. This is probably because we extracted each individual plant rather than a group of plants simultaneously. For segmentation of individual Chinese cabbages, we observed that adjacent cabbages that were not easily divided could be segmented by the model. Each segmented cabbage was then used to predict the width, length, and SPAD value.

For width prediction, the *R*^2^ values for the multispectral image UNet model, the RGB image UNet model without resolution enhancement, and the RGB image UNet model with resolution enhancement were 0.8824, 0.8235, and 0.8434, respectively. The RMSPEs were 0.0659, 0.1051, and 0.0900, and the RMSEs were 3.7258, 5.2984, and 5.0982. For length prediction, the *R*^2^ values of the 3 models were 0.8877, 0.7591, and 0.8977, the RMSPEs were 0.0755, 0.1037, and 0.2419, and the RMSEs were 5.6350, 7.7459, and 3.7227. The width prediction performance of the RGB model with SRGAN enhancement was inferior to that of the multispectral model, but its length prediction performance was the highest (Fig. 10). We also observed that length prediction was more accurate than width prediction. This may be because the cabbages were grown in rows, and the row spacing was large. The segmentation results were not good when portions of the cabbage widths partially overlapped within a row. The better length prediction of the RGB image model with SRGAN was due to the improved image resolution. In addition, the increased RMSPE of the RGB model with SRGAN was due to the prediction error of 1 of the measured samples, and the large error was generated by the segmentation result. Few studies have focused on plant width and length rather than plant height and biomass. Some researchers have studied the plant height of maize, wheat, and sorghum (*Sorghum bicolor* (L.) Moench) and established predictive models from radar data and UAV remote sensing images [40–43]. Their results also confirmed that UAV remote sensing could be used in breeding trials.

For SPAD prediction, the *R*^2^ value of the RGB image UNet model with SRGAN resolution enhancement was greater than 0.75. The *R*^2^ values of the multispectral image UNet model, the RGB UNet model without resolution enhancement, and the RGB UNet model with resolution enhancement were 0.7210, 0.7034, and 0.7978, respectively. The performance of the RGB model with SRGAN was the best, and the resolution enhancement may be the reason for its excellent segmentation performance. It is important to estimate leaf chlorophyll content accurately by remote sensing methods [15]. In a relevant study, the RMSE of SPAD prediction was greater than 2.30 [27], and the *R*^2^ value was greater than 0.85; the experimental equipment used in the study was a multispectral imaging device with 19 bands, which was costly. Jiang et al. [16] used a UAV-based multispectral camera and a random forest regression approach to predict SPAD values, and the RMSE of the predictive model was 2.5350 to 2.8610. By contrast, the RMSE in our research was between 1.7560 and 2.1710, demonstrating that the prediction of phenotypic information for Chinese cabbage was robust.

The ultimate goal of the present study was to select the best model for plant size and SPAD prediction in order to provide technical support for Chinese cabbage breeding. In the past, manual measurement of these parameters has been subjective and time-consuming, and reduced time requirement is a prerequisite for high-throughput phenotyping [13,44–46]. In this study, a UAV was used to investigate field traits in Chinese cabbage breeding. A UAV system equipped with a visible light sensor and a multispectral sensor was used to collect hundreds of high-resolution images for estimating width, length, and SPAD value. The prediction accuracy of 3 deep learning models based on an improved UNet semantic segmentation model was compared. For the validation set of the UNet model, the RGB image model with resolution enhancement showed the best performance. Individual cabbages could be separated from the complex land background by the UNet model. Regression analysis was performed on the predicted data and actual measurements. The prediction performance of the RGB image UNet model without enhanced resolution was poor, whereas resolution enhancement of the RGB image with SRGAN increased the identification accuracy for individual Chinese cabbages. In our work, the acquisition of drone imagery required about one-tenth the time of visual assessment, making it an efficient and inexpensive tool for rapid prediction of these characters in Chinese cabbage.

This study introduces a technical approach for the future investigation of Chinese cabbage field traits. In future studies, UAV images will be collected at different heights to improve the feasibility of SRGAN, and the model will be improved using large amounts of experimental data. Future research will study the influence of different topographies on model performance, and more traits such as plant height and Chinese cabbage holding pattern will be predicted as quickly as possible.

## Data Availability

The data used to support the findings of this study are available from the corresponding author Xiaofei Fan.
